# The early life nutritional environment and early life stress as potential pathways towards the metabolic syndrome in mid-life? A lifecourse analysis using the 1958 British Birth cohort

**DOI:** 10.1186/s12889-016-3484-0

**Published:** 2016-08-18

**Authors:** C. Delpierre, R. Fantin, C. Barboza-Solis, B. Lepage, M. Darnaudéry, M. Kelly-Irving

**Affiliations:** 1 INSERM, UMR1027, Toulouse, F-31000 France; 2 Université Toulouse III Paul-Sabatier, UMR1027, Toulouse, F-31000 France; 3 Universidad de Costa Rica, 2060 San José, Costa Rica; 4 Department of Epidemiology, Centre Hospitalier Universitaire de Toulouse, Toulouse, France; 5 Université Bordeaux, Laboratoire NUTRINEURO, UMR 1286, F-33076 Bordeaux, France; 6 INRA, Lab NUTRINEURO, UMR 1286, F-33076 Bordeaux, France

**Keywords:** Metabolic syndrome, Early nutritional exposure, Early psychosocial exposures, Cohort studies, Social epidemiology, Lifecourse

## Abstract

**Background:**

Lifecourse studies suggest that the metabolic syndrome (MetS) may be rooted in the early life environment. This study aims to examine the pathways linking early nutritional and psychosocial exposures and the presence of MetS in midlife.

**Methods:**

Data are from the National Child Development Study including individuals born during 1 week in 1958 in Great Britain and followed-up until now. MetS was defined based on the National Cholesterol Education Program Adult Treatment Panel III classification. Mother’s pre-pregnancy body mass index (BMI) was used as a proxy of the early nutritional environment and Adverse Childhood Experiences (ACE) as a proxy for early psychosocial stress. Socioeconomic characteristics, pregnancy and birth conditions were extracted as potential confounders. Adult health behaviors, BMI, socioeconomic environment and psychological state were considered as mediating variables.

Multivariate models were performed by including variables sequentially taking a lifecourse approach.

**Results:**

37.5 % of men and 19.8 % of women had MetS. Participants with an obese/overweight mother presented a higher risk of MetS than those whose mother had a normal pre-pregnancy BMI. Men exposed to two ACE or more, and women exposed to one ACE, were more at risk of MetS compared to unexposed individuals. After including confounders and mediators, mother’s pre-pregnancy BMI was still associated with MetS in midlife but the association was weakened after including participant’s adult BMI. ACE was no longer associated with MetS after including confounders in models.

**Conclusions:**

The early nutritional environment, represented by mother’s pre-pregnancy BMI, was associated with the risk of MetS in midlife. An important mechanism involves a mother-to-child BMI transmission, independent of birth or perinatal conditions, socioeconomic characteristics and health behaviors over the lifecourse. However this mechanism is not sufficient for explaining the influence of mother’s pre-pregnancy BMI which implies the need to further explore other mechanisms in particular the role of genetics and early nutritional environment. ACE is not independently associated with MetS. However, other early life stressful events such as emergency caesarean deliveries and poor socioeconomic status during childhood may contribute as determinants of MetS.

## Background

Metabolic syndrome (MetS) is a cluster of factors associated with a higher risk of type 2 diabetes, cardiovascular diseases and mortality [1, 2]. Although various definitions exist for MetS, its component factors generally include central obesity, dyslipidemia (elevated triglycerides, low high-density lipoproteins), hypertension and hyperglycemia. The prevalence of MetS is estimated at around 30 % in the US or in Europe for adult mainly above 30 years [3, 4] and it is likely to increase alongside the high frequency of related factors including obesity, sedentary lifestyles and unbalanced diets. Moreover recent evidence suggests a link between MetS and various chronic diseases such as cancer, notably through proinflammatory mechanisms and hormonal processes [3, 5, 6], but also cognitive decline [7] and dementia [8] through metabolic and vascular mechanisms. MetS is therefore potentially associated with a wide range of chronic diseases.

It has been suggested that MetS could be partly rooted in early life environments [9] and therefore taking a lifecourse perspective to understanding the development of MetS is relevant. Lifecourse studies have shown a relationship between socioeconomic position during childhood, measured retrospectively or prospectively, and the body mass index (BMI), ischaemic heart disease [10, 11] as well as MetS itself during adulthood [12–15]. One of the proposed pathways by which early socioeconomic conditions could influence MetS involves the early nutritional environment. In fact parental BMI, in particular maternal BMI, has been associated with offspring’s adiposity and BMI. Individuals whose parents had a higher BMI during their childhoods were more likely to be obese in adulthood [16]. Such lifecourse studies specifically on MetS are rarer, but evidence exists for a link between parental BMI and the subsequent risk for offspring to have higher levels of some components of MetS such as an atherosclerotic lipid profile [17]. This association seems to be explained only partially by adult lifestyles and lifetime socioeconomic position [16, 17]. Potential explanations for this parent–child BMI transmission may include biological mechanisms operating through genetic predisposition, prenatal “programming” during intrauterine and early development that constitute critical windows of development particularly sensitive to environmental challenges through activation of the stress axis and metabolic modifications involving epigenetic changes [18, 19], or shared environments such as cultural and familial eating patterns [20]. Whatever the mechanisms involved in the parent–child BMI transmission, they deserve to be further studied.

In addition to early nutritional exposures, another pathway by which early socioeconomic conditions could influence MetS involves early psychosocial stress. To study early life stress in human populations, adverse childhood experiences (ACE), that may be defined as a set of traumatic psychosocial conditions not under the child’s control that tend to co-occur usually before the age of 16, causing chronic or acute stress responses which may alter fundamental biological functions [21] like trauma abuse or maltreatment in childhood, have been used. Although definitions may vary, ACE has been identified as a factor influencing global physiological functioning in adulthood [22], impacting global health in particular cardiovascular health [23–25]. A large majority of such studies use retrospective measures for ACE some studies have also used a prospective measure and found it to be related to premature mortality [26], cancer risk [27] or cardiovascular health [28–30]. A number of mechanisms from animal to population studies linking early life stress and subsequent risk of symptoms composing MetS like obesity or glycemia have been proposed [31, 32] however, these pathways deserve to be examined in more detail.

An important remaining issue is to disentangle common and separate pathways by which both early nutritional and psychosocial stress can influence MetS. In fact, few studies combine both early nutritional and psychosocial stress as determinants of MetS. However biological links between nutrition and stress have been identified [33] and some common pathways may exist for these two exposures [34]. Identifying the respective role of early nutritional and psychosocial environment on the subsequent risk of MetS involves implementing studies to analyze the joint influence of these two exposures using a comprehensive lifecourse approach to take confounders into account and identify potential pathways.

Our study aims to investigate the pathways linking both early nutritional and psychosocial exposures to the presence of the MetS in midlife in the 1958 British birth cohort study taking a lifecourse approach. Three main pathways have been tested: (i) a socioeconomic/materialist pathway, (ii) a health behaviors pathway, (iii) a psychological pathway.

## Method

**Fig. 1:**
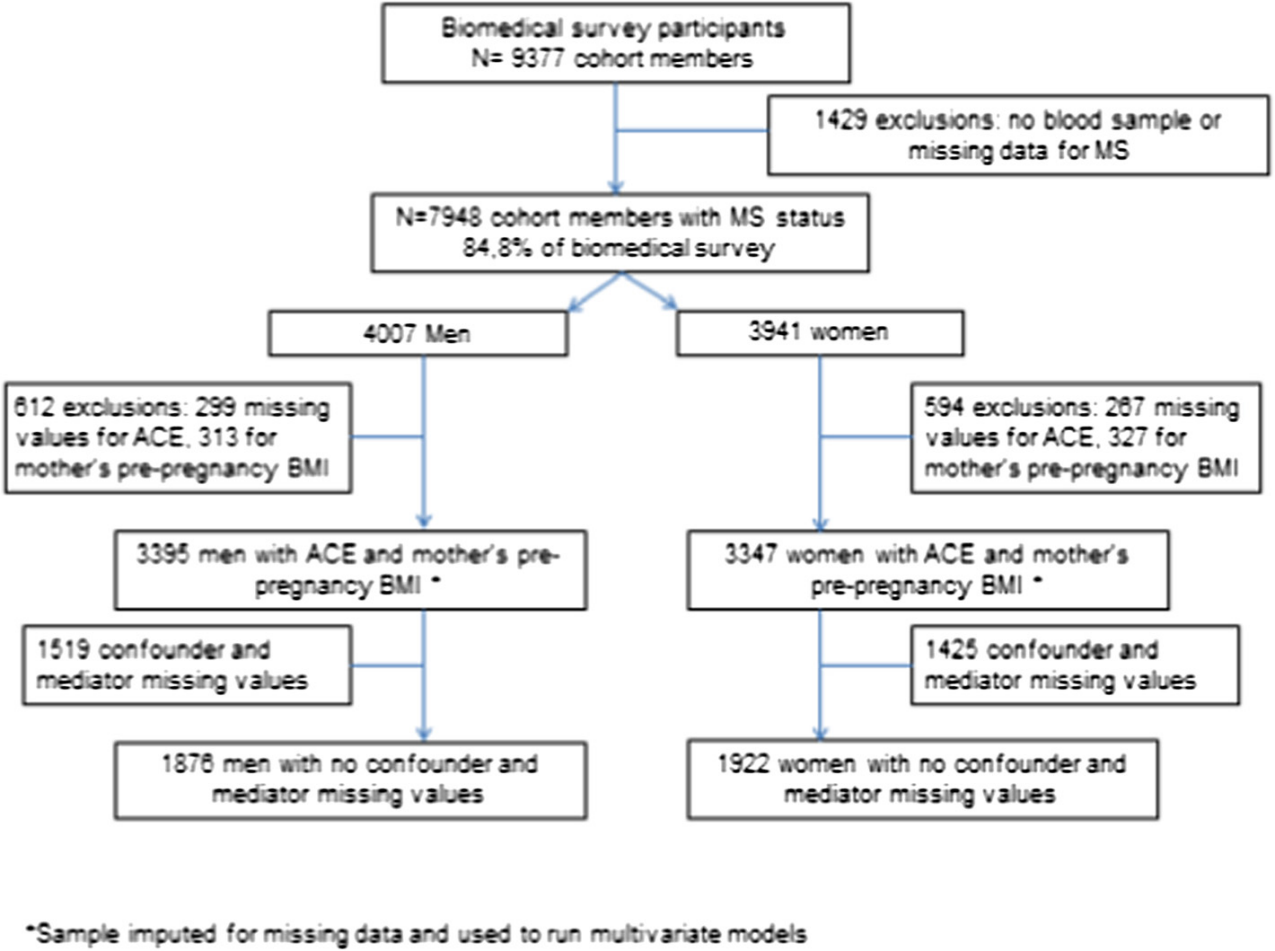
Flow chart showing the sample selection

This study used data from the 1958 National Child Development Study (NCDS) which included all births during 1 week in March 1958 (*n* = 18 558) in Great Britain. Subsequent data collections were carried out on cohort members aged 7, 11, 16, 23, 33, 42, 46 and 50 years. The NCDS has been described in detail elsewhere [35]. A biomedical survey (9377 cohort members participating) was conducted when participants were aged 44–46 years. The flow chart corresponding to the sample selection used for this study is presented in Fig. 1.

Our study sample consisted of 3395 men and 3347 women with data on MetS and our two main exposure variables. The complete sample consisted respectively of 1876 men and 1922 women by defining complete cases as people with complete data for all the covariates.

### Outcome variable

MetS was defined based on the NCEP-ATP III (National Cholesterol Education Program Adult Treatment Panel III) clinical criteria except for plasma glucose, which was not recorded in the NCDS biomedical survey and replaced here by using glycosylated hemoglobin (HbA1c) ≥6.5 %. MetS was identified at the age of 44–46 years old if three or more of the following occurred: blood pressure > =130/85 mmHg; High Density Lipoprotein <0.40 g/L for men and <0.50 g/l for women; Triglycerides > =150 mg/dL; waist circumference >88 cm for women and > 102 cm for men; HbA1c ≥6.5 % [36].

### Exposure variables

Maternal BMI before pregnancy was used as a proxy of early nutritional stress, as an indicator of a high calorie/fat nutritional environment during pregnancy which has been identified as a risk factor of subsequent metabolic diseases [9]. Mother’s pre-pregnancy weight, that was self-reported at birth, and her height measured after the birth were used to construct maternal BMI before pregnancy and was grouped as follows: <18.5 kg/m2 for underweight, 18.5–24.9 for normal, 25–29.9 for overweight and ≥30 kg/m2 for obese adults.

Adverse childhood experiences (ACE) were used as a proxy of early psychosocial stress. Our measure of ACE has been presented in details in previous works [26, 27]. From previous works on ACE, specifically work conducted by a WHO expert committee in 2009 [37], we constructed a theoretical framework prior to extracting any data, in order to create a measurement with robust content validity. We have identified ACE as a set of traumatic and stressful psychosocial conditions that are out of the child’s control, that tend to co-occur and often persist over time. We have restricted ACE to intra-familial events or conditions in the child’s immediate environment causing chronic stress responses. From variables prospectively collected at age 7, 11, 16 by the child’s parent or their teacher, exposure to adversity was identified by a positive response to any of the following categories:

1. Child in care: child has ever been in public/voluntary care services or foster care at age 7, 11 or 16;
2. Physical neglect: child appears undernourished/dirty aged 7 or 11, information collected from the response from child’s teacher to the Bristol Social Adjustment Guide;
3. Offenders: The child lived in a household where a family member (person living in the same household as the child) was in prison or on probation (age 11 years) or is in contact with probation service at 7 or 11 years; the child has ever been to prison or been on probation at 16 years;
4. Parental separation: The child has been separated from their father or mother due to death, divorce, or separation at 7, 11 or 16 years;
5. Mental illness: Household has contact with mental health services at 7 or 11 years; Family member has mental illness at 7 and 11 or 16 years;
6. Alcohol abuse: Family member has alcohol abuse problem at 7 years.

A three category variable was then constructed by counting the reports of adversities (0 adversities/1 adversity/2or + adversities).

Respondents were excluded if they had missing data for all six categories. Respondents were considered as having no adversities if they answered ‘no’ all the categories or if they answered ‘no’ to one or more category and the other categories were missing.

### Covariates

Potential *confounders* for the associations between the two main exposures studied (mother’s pre-pregnancy BMI and ACE) and MetS were selected a priori based on literature including recent work we performed on MetS during adulthood on the same cohort [38]. Selected variables concerned early life socioeconomic conditions: household and parental characteristics [mother’s education level (left school at 15 y or later/before 14 y), mother’s partner’s (or father’s if unavailable) social class (non-manual/manual), overcrowded household (people per room >1.5 or ≤1.5)]. To capture elements in the early environment potentially related to stressful socioeconomic or psychosocial conditions and subsequent MetS, we selected proxy variables on birth and perinatal conditions [38]: maternal smoking during pregnancy (no smoking, sometimes, moderately, heavily), type of delivery (vaginal, emergency caesarean, elective caesarean), Mother’s parity in 1958, maternal age at birth (divided according quartiles), birthweight (divided according quartiles) and gestational age (<38, 38, 39–41 and >41 weeks) collected at birth; breastfed collected at 7 years (no, yes for less than 1 month, yes more than 1 month) .

Potential *adult mediating factors* were also selected to explore three main pathways:

i) a socioeconomic/materialist one, including respondent’s educational attainment at 23 years (A level/O level/no qualification), respondent’s occupational social class at 33 years (non-manual/manual active), wealth based on information about home ownership and the price of the house adjusted for economic inflation of the year of purchase and then divided in quartiles (not owner/Q1—owner lowest price/owner-Q2/owner-Q3/owner-Q4);
ii) a health behaviors pathway including physical activity [physically active/moderately active/inactive], alcohol consumption [abstainers (reported not consuming any alcohol in the previous week)/moderate (women: between 1 and 14 units in the previous week; men: between 1 and 21 units in the previous week) /heavy drinkers (women: >14 units in the previous week; men: >21 units in the previous week)], smoking status [nonsmoker/former smoker/smoker (<10 cigarettes/smoker; 10–19 cigarettes/smoker; >20 cigarettes)] and BMI (normal/underweight/overweight/obese) at 23 years. Adult life style variables are available at other points along the lifecourse, however in our model, these adult variables at the age 23 are considered as a proxy of behavioural patterns in early adulthood and controlling for them serve as a first step to understanding possible mechanism.
iii) a psychological one including the Rutter’s malaise inventory that measures psychological distress at 23 years [39, 40]. It comprises 24 binary items on both emotional and somatic symptoms, the individual being considered as having a psychological malaise if s/he reported experiencing more than 7 out of 24 symptoms (no malaise/malaise); and marital status at 33 y (couple/single/divorced or widowed).

### Statistical analyses

Sample characteristics were first described. Bivariate crosstabulations were done between our two main exposures, mother’s pre-pregnancy BMI and ACE, and the subsequent risk of MetS. Then data were analyzed in multivariate models. A series of logistic regression models were performed taking a lifecourse perspective: Model 1 included mother’s pre-pregnancy BMI and all variables measured at or around birth; in Model 2 we added ACE measured between 7 and 16 years old; in Model 3 we then added potential mediators measured at 23 years except for participants’ BMI at 23 years; in Model 4 we included all variables measured at 23 years including participant’s BMI; finally, in Model 5 we added variables measured at 33 years corresponding to the full model.

We determined the respective statistical contributions of confounders and mediators in explaining the association between pre pregnancy maternal BMI or ACE and MetS by using a traditional approach to mediation analyses [41]. We calculated the percentage attenuation in the β coefficient for maternal pre-pregnancy BMI and adversity after inclusion of confounders and mediators in models using the following formula:

$$ 100\times \left(\beta ref\kern0.5em  model-\beta ref\kern0.5em  model+ counfounders/ mediators\right)/\left(\beta ref\kern0.5em  model\right) $$

This approach has been used elsewhere to explore the influence of social determinants on health inequalities [12, 42, 43]. A 95 % CI was then calculated around the percentage attenuation using a bootstrap method with 1000 re-samplings. When the 95 %CI did not include 0, the attenuation was considered as significant.

All the analyses were performed separately for men and women given that sex/gender differences regarding biological pathways towards MetS having been shown, notably in the same study [44].

To control for possible bias due to missing data, multivariate analyses were performed using imputed data for covariates with missing data using the multiple imputation program ICE in STATA V.11. Ten imputations were conducted taking the missing-at-random (MAR) assumption. Each covariate with missing values was imputed including in the imputation model all confounders and mediators of the multivariate model as well as variables from other sweeps correlated with the variable to impute. These variables from other sweeps were selected a priori because they could improve the imputation model estimations and we keep only those that achieved the 5 % significance cutoff. For example, we imputed self-reported alcohol consumption at 23 y using information on the individual drinking at 33 and 42 years. Neither the exposure variables of interest (maternal BMI before pregnancy and ACE) not the MetS variable were included in the multiple imputation.

Sensitivity analyses were performed by replicating analyses on complete cases.

## Results

The sample characteristics are presented in Table 1. Among men, 19.1 and 4.2 % of their mothers were overweight or obese before pregnancy respectively. These proportions were similar in women (19.6 % and 3.8 % for overweight and obese mother respectively). Regarding ACE, 20.4 % of men and 19.8 % of women had been exposed to one adversity during their childhood, 6.5 % of men and 6.4 % of women being exposed to at least two adversities.

**Table 1:** Characteristics of the studied sample

	Men (*N* = 3395)		Women (*N* = 3347)	
	Number	Percent	Number	Percent
Metabolic syndrome				
No	2121	62.5	2685	80.2
Yes	1274	37.5	662	19.8
Metabolic syndrome components				
High systolic blood pressure	1861	54.8	828	24.7
High diastolic blood pressure	1261	37.1	593	17.7
Low HDL cholesterol	286	8.4	429	12.8
High triglycerides	2115	62.3	1097	32.8
High waist circumference	1090	32.1	1188	35.5
High HbA1c	101	3.0	61	1.8
Mother’s BMI				
Normal	2 474	72.9	2 396	71.6
Underweight	129	3.8	167	5.0
Overweight	648	19.1	656	19.6
Obese	144	4.2	128	3.8
Adverse childhood experiences				
None	2 482	73.1	2 471	73.8
One	693	20.4	662	19.8
Two or more	220	6.5	214	6.4
Mother’s education level				
Left school at 15 or later	921	27.1	903	27.0
Left school before 14	2 453	72.3	2 431	72.6
Missing	21	0.6	13	0.4
Father’s social class at birth				
Non-manual	988	29.1	922	27.5
Manual	2 271	66.9	2 274	67.9
Missing	136	4.0	151	4.5
Overcrowding				
> 1.5 person/room	360	10.6	408	12.2
< =1.5 person/room	2 949	86.9	2 870	85.7
Missing	86	2.5	69	2.1
Birthweight				
Q1 - Low weight	711	20.9	727	21.7
Q2	935	27.5	871	26.0
Q3	871	25.7	829	24.8
Q4 - High weight	763	22.5	828	24.7
Missing	115	3.4	92	2.7
Mother smoked during pregnancy				
No	2 301	67.8	2 225	66.5
Sometimes	212	6.2	196	5.9
Moderately	479	14.1	507	15.1
Heavily	367	10.8	385	11.5
Missing	36	1.1	34	1.0
Mother’s age at birth				
23 years or less	866	25.5	889	26.6
24 to 27 years	974	28.7	924	27.6
28 to 31 years	770	22.7	732	21.9
32 years or more	784	23.1	799	23.9
Missing	1	0.0	3	0.1
Breastfed				
No	956	28.2	922	27.5
Yes, for less than 1 month	757	22.3	756	22.6
Yes, more than 1 month	1 477	43.5	1 482	44.3
Missing	205	6.0	187	5.6
Parity				
Oldest child	1 221	36.0	1 255	37.5
2nd child	1 151	33.9	1 016	30.4
3rd child or more	1 023	30.1	1 076	32.1
38 weeks or less	275	8.1	261	7.8
Gestational age				
38 weeks or less	275	8.1	261	7.8
39 to 41 weeks	1 970	58.0	1 893	56.6
42 weeks or more	855	25.2	875	26.1
Missing	295	8.7	318	9.5
Type of delivery				
Vaginal	3 302	97.3	3 271	97.7
Emergency caesarean	51	1.5	44	1.3
Elective caesarean	42	1.2	32	1.0
Smoking status at 23				
Non-smoker	858	25.3	991	29.6
Former smoker	928	27.3	840	25.1
Smoker - Less than 10 cig.	201	5.9	293	8.8
Smoker - 10 to 19 cig.	398	11.7	430	12.8
Smoker - More than 20 cig.	529	15.6	408	12.2
Missing	481	14.2	385	11.5
Alcohol consumption at 23				
Moderate	1 412	41.6	1 567	46.8
Abstainers	346	10.2	1 031	30.8
Heavy drinkers	1 155	34.0	363	10.8
Missing	482	14.2	386	11.5
Physical activity at 23				
Physically active	1 254	36.9	730	21.8
Moderately active	542	16.0	420	12.5
Inactive	1 114	32.8	1 812	54.1
Missing	485	14.3	385	11.5
Malaise inventory at 23				
No	2 815	82.9	2 677	80.0
Yes	94	2.8	282	8.4
Missing	486	14.3	388	11.6
Education level at 23				
Passed A levels	731	21.5	682	20.4
Passed O levels	1 119	33.0	1 342	40.1
No qualifications	1 062	31.3	936	28.0
Missing	483	14.2	387	11.6
BMI at 23				
Normal	2 299	67.7	2 377	71.0
Underweight	68	2.0	183	5.5
Overweight	447	13.2	300	9.0
Obese	52	1.5	73	2.2
Missing	529	15.6	414	12.4
Occupational social class at 33				
Non-manual	1 518	44.7	1 981	59.2
Manual	1 314	38.7	850	25.4
Missing	563	16.6	516	15.4
Wealth at 33				
Not owner	672	19.8	672	20.1
Owner - Q1 (Low price)	561	16.5	570	17.0
Owner - Q2	569	16.8	591	17.7
Owner - Q3	594	17.5	566	16.9
Owner - Q4	544	16.0	588	17.6
Missing	455	13.4	360	10.8
Marital status at 33				
Couple	2 396	70.6	2 496	74.6
Single	417	12.3	276	8.2
Divorced or widowed	144	4.2	254	7.6
Missing	438	12.9	321	9.6

MetS was frequent, in particular in men among whom 37.5 % had MetS versus 19.8 % of women. This difference was mainly due to a higher proportion of high blood pressure in men as a higher proportion of triglycerides > =150 mg/dL, the proportion of high level for the other components of MetS being close in men and women (Table 1). MetS proportions increased in men and women gradually as mother’s pre pregnancy BMI increased (Table 2). In men, compared to the proportion (35.7 %) of MetS when their mothers had a normal BMI before pregnancy, 42.3 % had MetS when their mothers were overweight before pregnancy (crude OR = 1.32, 95 % CI: 1.11–1.57) and 47.2 % when their mothers were obese before pregnancy (crude OR = 1.61, 95 % CI: 1.15–2.26). In women, compared to the proportion (17.9 %) of MetS when their mothers had a normal BMI before pregnancy, 25.0 % had MetS when their mothers were overweight before pregnancy (crude OR = 1.53, 95 % CI: 1.25–1.88) and 29.7 % had MetS when their mothers were obese before pregnancy (crude OR = 1.94, 95 % CI: 1.31–2.88).

**Table 2:** Proportion and odds of having the metabolic syndrome according to mother’s pre-pregnancy BMI and childhood adversity

	Metabolic syndrome			
	Men (*N* = 3395)			
	N (%)	*p* value	OR (95 %CI)	*p* value
Mother’s pre-pregnancy BMI				
Normal	883 (35.7)	0.001	1	
Underweight	49 (38)		1.10 (0.77–1.59)	0.596
Overweight	274 (42.3)		1.32 (1.11–1.57)	0.002
Obese	68 (47.2)		1.61 (1.15–2.26)	0.006
Adverse childhood experiences				
None	922 (37.1)	0.06	1	
One	253 (36.5)		0.97 (0.82–1.16)	0.76
Two or more	99 (45.0)		1.38 (1.05–1.83)	0.02
	Women (*N* = 3347)			
	N (%)	*p* value	OR (95 %CI)	*p* value
Mother’s pre-pregnancy BMI				
Normal	428 (17.9)	<0.001	1	
Underweight	32 (19.2)		1.09 (1.73–1.62)	0.673
Overweight	164 (25.0)		1.53 (1.25–1.88)	<0.001
Obese	38 (29.7)		1.94 (1.31–2.88)	<0.001
Adverse childhood experiences				
None	463 (18.7)	0.04	1	
One	151 (22.8)		1.28 (1.04–1.58)	0.02
Two or more	48 (22.4)		1.25 (0.90–1.76)	0.19

MetS was also associated with ACE but not on a gradient. In men, 45 % of those exposed to at least two adversities during childhood had MetS compared to 37.1 % in non-exposed men (crude OR = 1.38, 95 % CI: 1.05–1.83). In women, 22.8 % of those exposed to one adversity during childhood had MetS compared to 18.7 % in non-exposed women (crude OR = 1.28, 95 % CI: 1.04–1.58).

**Fig. 2:**
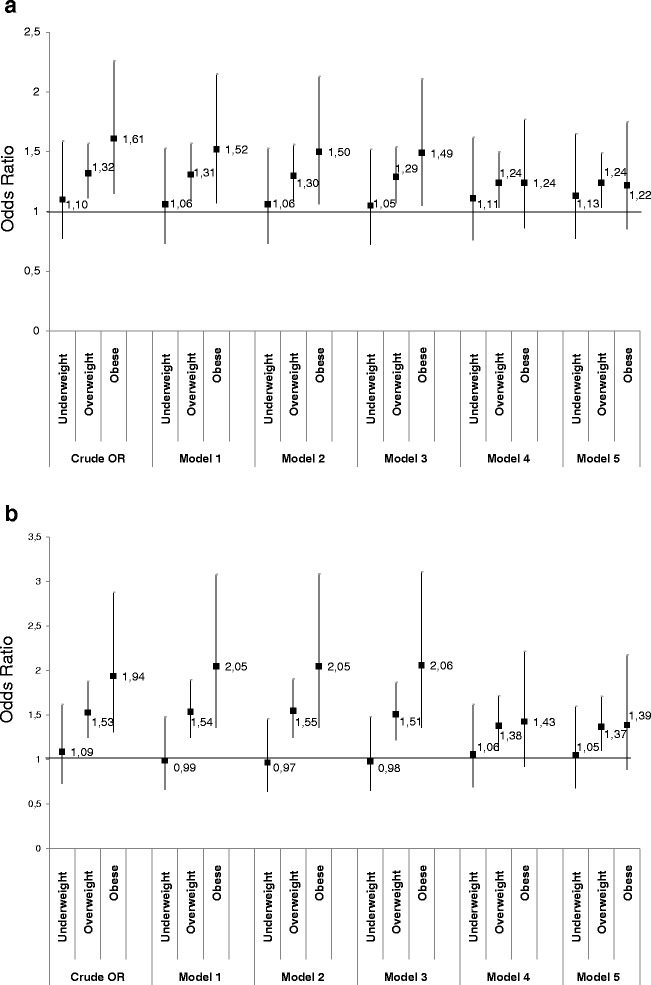
**a** Association between mother’s pre-pregnancy BMI and metabolic syndrome in men according to models **b** Association between mother’s pre-pregnancy BMI and metabolic syndrome in women according to models

Results of multivariate analyses are presented in Tables 3, 4 and in Fig. 2. In men (Table 3, Fig. 2a) the link between mother’s pre-pregnancy BMI and MetS persisted after adjustment for perinatal confounders (model 1), as after inclusion of ACE (model 2). The inclusion in the model of potential mediators at 23 years did not change the strength of this association (model 3) except when participants’ BMI at 23 years was added (model 4). Compared with model 1, after adding participant’s BMI at 23 years, the association between mother’s pre-pregnancy BMI and MetS was attenuated by 54.7 % (95 % CI: 25.4–170.7 %) for obese (OR = 1.24, 95 % CI: 0.86–1.77) and by 21.4 % (95 % CI: 0.3–78.8 %) for overweight mother’s before pregnancy (OR = 1.24, 95 % CI: 1.03–1.50) respectively. Including variables at 33 years (model 5) did not change the association further.

**Table 3:** Odds of having metabolic syndrome. Lifecourse multivariate logistic regression models using data from multiple imputations. Men (*N* = 3395)

	Model 1			Model 2			Model 3			Model 4			Model 5		
	OR	CI 95 %	p	OR	CI 95 %	p	OR	CI 95 %	p	OR	CI 95 %	p	OR	CI 95 %	p
Mother’s BMI															
Normal	1			1			1			1			1		
Underweight	1.06	(0.73–1.53)	0.760	1.06	(0.73–1.53)	0.771	1.05	(0.72–1.52)	0.798	1.11	(0.76–1.62)	0.599	1.13	(0.77–1.65)	0.533
Overweight	1.31	(1.09–1.57)	0.003	1.30	(1.08–1.56)	0.004	1.29	(1.07–1.54)	0.007	1.24	(1.03–1.50)	0.024	1.24	(1.03–1.49)	0.025
Obese	1.52	(1.07–2.15)	0.018	1.50	(1.06–2.13)	0.022	1.49	(1.05–2.11)	0.026	1.24	(0.86–1.77)	0.244	1.22	(0.85–1.75)	0.282
Mother’s education level															
Left school at 15 or later	1			1			1			1			1		
Left school before 14	1.38	(1.16–1.65)	<0.001	1.38	(1.16–1.65)	<0.001	1.36	(1.13–1.63)	0.001	1.34	(1.11–1.61)	0.002	1.34	(1.11–1.61)	0.002
Father’s social class at birth															
Non-manual	1			1			1			1			1		
Manual	1.25	(1.05–1.49)	0.013	1.25	(1.05–1.49)	0.013	1.23	(1.02–1.47)	0.029	1.17	(0.98–1.41)	0.087	1.18	(0.98–1.42)	0.082
Overcrowding															
*> 1.5 personne/chambre*	1			1			1			1			1		
*< =1.5 personne/chambre*	0.97	(0.77–1.22)	0.775	0.97	(0.77–1.22)	0.789	0.98	(0.77–1.24)	0.842	0.97	(0.76–1.24)	0.805	0.98	(0.77–1.25)	0.882
Birthweight															
Q1 - Low weight	1			1			1			1			1		
Q2	0.87	(0.70–1.07)	0.187	0.87	(0.71–1.08)	0.204	0.88	(0.71–1.09)	0.229	0.87	(0.70–1.08)	0.207	0.88	(0.71–1.09)	0.239
Q3	1.02	(0.82–1.27)	0.832	1.03	(0.83–1.28)	0.800	1.04	(0.83–1.29)	0.741	1.01	(0.80–1.26)	0.961	1.01	(0.81–1.27)	0.900
Q4 - High weight	0.90	(0.72–1.14)	0.390	0.91	(0.73–1.15)	0.430	0.92	(0.73–1.16)	0.471	0.84	(0.66–1.07)	0.151	0.84	(0.66–1.07)	0.161
Mother smoked during pregnancy															
No	1			1			1			1			1		
Sometimes	1.16	(0.87–1.56)	0.308	1.16	(0.86–1.55)	0.328	1.14	(0.85–1.53)	0.389	1.07	(0.79–1.45)	0.644	1.07	(0.79–1.45)	0.645
Moderately	1.12	(0.91–1.38)	0.268	1.12	(0.91–1.38)	0.272	1.12	(0.91–1.38)	0.286	1.06	(0.86–1.31)	0.594	1.06	(0.86–1.31)	0.576
Heavily	0.98	(0.78–1.24)	0.878	0.97	(0.77–1.23)	0.824	0.95	(0.75–1.20)	0.639	0.90	(0.70–1.14)	0.371	0.89	(0.70–1.14)	0.361
Mother’s age at birth															
23 years or less	1			1			1			1			1		
24 to 27 years	1.09	(0.90–1.33)	0.377	1.09	(0.90–1.33)	0.381	1.10	(0.90–1.35)	0.335	1.11	(0.91–1.36)	0.300	1.12	(0.91–1.37)	0.274
28 to 31 years	1.01	(0.82–1.26)	0.894	1.02	(0.82–1.27)	0.879	1.04	(0.83–1.30)	0.719	1.05	(0.83–1.31)	0.697	1.04	(0.83–1.31)	0.712
32 years or more	0.83	(0.66–1.05)	0.119	0.84	(0.66–1.06)	0.134	0.86	(0.68–1.09)	0.209	0.85	(0.66–1.08)	0.179	0.85	(0.66–1.08)	0.182
Breastfed															
No	1			1			1			1			1		
Yes, for less than 1 month	1.02	(0.83–1.24)	0.877	1.01	(0.83–1.24)	0.887	1.01	(0.83–1.24)	0.913	1.02	(0.83–1.26)	0.852	1.02	(0.83–1.26)	0.863
Yes, more than 1 month	1.00	(0.84–1.19)	0.993	1.00	(0.84–1.19)	0.995	1.01	(0.84–1.20)	0.934	1.03	(0.86–1.22)	0.781	1.03	(0.86–1.23)	0.780
Parity															
Oldest child	1			1			1			1			1		
2nd child	0.88	(0.74–1.05)	0.168	0.88	(0.74–1.05)	0.159	0.87	(0.73–1.04)	0.132	0.88	(0.73–1.05)	0.165	0.87	(0.73–1.05)	0.148
3rd child or more	1.00	(0.82–1.23)	0.965	1.00	(0.81–1.22)	0.973	0.97	(0.78–1.19)	0.747	0.99	(0.80–1.23)	0.946	0.99	(0.80–1.23)	0.924
Gestational age															
38 weeks or less	1			1			1			1			1		
39 to 41 weeks	1.19	(0.89–1.60)	0.248	1.19	(0.88–1.60)	0.253	1.21	(0.90–1.62)	0.215	1.17	(0.87–1.57)	0.295	1.18	(0.87–1.60)	0.284
42 weeks or more	1.18	(0.85–1.62)	0.323	1.17	(0.85–1.61)	0.331	1.18	(0.86–1.63)	0.299	1.17	(0.86–1.61)	0.318	1.19	(0.86–1.63)	0.297
Type of delivery															
Vaginal	1			1			1			1			1		
Emergency caesarean	1.86	(1.06–3.29)	0.032	1.83	(1.03–3.23)	0.038	1.83	(1.03–3.25)	0.038	1.98	(1.11–3.56)	0.022	2.02	(1.13–3.63)	0.018
Elective caesarean	0.73	(0.37–1.44)	0.359	0.73	(0.37–1.44)	0.361	0.77	(0.39–1.54)	0.465	0.75	(0.37–1.50)	0.412	0.75	(0.37–1.51)	0.423
Adverse childhood experiences															
None				1			1			1			1		
One				0.93	(0.78–1.12)	0.452	0.91	(0.76–1.09)	0.296	0.91	(0.76–1.10)	0.331	0.91	(0.76–1.10)	0.341
Two or more				1.19	(0.89–1.59)	0.243	1.12	(0.83–1.50)	0.469	1.18	(0.87–1.59)	0.294	1.16	(0.86–1.57)	0.340
Smoking status at 23															
Non-smoker							1			1			1		
Former smoker							0.95	(0.78–1.15)	0.575	0.94	(0.77–1.15)	0.541	0.94	(0.77–1.14)	0.521
Smoker - Less than 10 cig.							1.13	(0.82–1.56)	0.438	1.15	(0.82–1.60)	0.414	1.15	(0.82–1.60)	0.424
Smoker - 10 to 19 cig.							0.95	(0.74–1.22)	0.685	0.98	(0.76–1.27)	0.883	0.97	(0.76–1.26)	0.839
Smoker - More than 20 cig.							1.11	(0.88–1.40)	0.378	1.13	(0.90–1.44)	0.295	1.13	(0.89–1.44)	0.304
Alcohol consumption at 23															
Moderate							1			1			1		
Abstainers							1.00	(0.76–1.31)	0.991	1.02	(0.77–1.35)	0.893	0.99	(0.75–1.31)	0.937
Heavy drinkers							1.08	(0.91–1.27)	0.368	1.06	(0.90–1.26)	0.497	1.06	(0.89–1.25)	0.521
Physical activity at 23															
Physically active							1			1			1		
Moderately active							1.13	(0.91–1.40)	0.259	1.13	(0.91–1.40)	0.283	1.12	(0.90–1.40)	0.297
Inactive							1.25	(1.04–1.52)	0.020	1.25	(1.03–1.53)	0.026	1.25	(1.02–1.52)	0.029
Malaise inventory at 23															
No							1			1			1		
Yes							1.30	(0.87–1.94)	0.200	1.28	(0.85–1.94)	0.237	1.30	(0.86–1.97)	0.218
Education level at 23															
Passed A levels							1			1			1		
Passed O levels							1.04	(0.84–1.31)	0.705	0.98	(0.79–1.23)	0.893	1.00	(0.78–1.28)	0.991
No qualifications							1.12	(0.88–1.43)	0.356	0.99	(0.77–1.26)	0.913	1.00	(0.75–1.34)	0.986
BMI at 23															
Normal										1			1		
Underweight										0.52	(0.28–0.96)	0.036	0.50	(0.27–0.93)	0.028
Overweight										2.27	(1.83–2.82)	<0.001	2.27	(1.83–2.82)	<0.001
Obese										3.52	(1.92–6.46)	<0.001	3.43	(1.87–6.31)	<0.001
Occupational social class at 33															
Non-manual													1		
Manual													0.94	(0.77–1.14)	0.516
Wealth at 33															
Not owner													1		
Owner - Q1 (Low price)													0.79	(0.62–1.01)	0.062
Owner - Q2													0.83	(0.65–1.06)	0.131
Owner - Q3													0.83	(0.64–1.07)	0.145
Owner - Q4													0.78	(0.60–1.02)	0.066
Marital status at 33															
Couple													1		
Single													0.96	(0.76–1.20)	0.709
Divorced or widowed													0.69	(0.47–1.00)	0.050

Model 1 included mother’s pre-pregnancy BMI and all variables measured at or around birth; Model 2: Model 1 + ACE; Model 3: Model 2 + potential mediators measured at 23 years except for participants’ BMI at 23 years; Model 4: Model 3 + participant’s BMI at 23 years; Model 5: Full model

**Table 4:** Odds of having metabolic syndrome. Lifecourse multivariate logistic regression models using data from multiple imputations. Women (*N* = 3347)

	Model 1			Model 2			Model 3			Model 4			Model 5		
	OR	CI 95 %	p	OR	CI 95 %	p	OR	CI 95 %	p	OR	CI 95 %	p	OR	CI 95 %	p
Mother’s BMI															
Normal	1			1			1			1			1		
Underweight	0.99	(0.66–1.48)	0.946	0.97	(0.64–1.46)	0.879	0.98	(0.65–1.48)	0.933	1.06	(0.69–1.62)	0.804	1.05	(0.68–1.60)	0.838
Overweight	1.54	(1.25–1.90)	<0.001	1.55	(1.25–1.91)	<0.001	1.51	(1.22–1.87)	<0.001	1.38	(1.10–1.72)	0.005	1.37	(1.10–1.71)	0.005
Obese	2.05	(1.36–3.08)	<0.001	2.05	(1.36–3.09)	<0.001	2.06	(1.36–3.11)	<0.001	1.43	(0.92–2.22)	0.116	1.39	(0.89–2.18)	0.145
Mother’s education level															
Left school at 15 or later	1			1			1			1			1		
Left school before 14	1.38	(1.10–1.73)	0.006	1.37	(1.09–1.72)	0.007	1.29	(1.02–1.63)	0.037	1.32	(1.04–1.69)	0.024	1.31	(1.03–1.68)	0.029
Father’s social class at birth															
Non-manual	1			1			1			1			1		
Manual	1.53	(1.22–1.94)	<0.001	1.52	(1.20–1.92)	<0.001	1.43	(1.13–1.82)	0.003	1.36	(1.06–1.75)	0.015	1.33	(1.04–1.71)	0.026
Overcrowding															
> 1.5 personne/chambre	1			1			1			1			1		
< =1.5 personne/chambre	1.07	(0.82–1.40)	0.593	1.08	(0.83–1.40)	0.586	1.09	(0.84–1.43)	0.508	1.11	(0.84–1.47)	0.469	1.12	(0.84–1.48)	0.444
Birthweight															
Q1 - Low weight	1			1			1			1			1		
Q2	0.78	(0.60–1.00)	0.049	0.77	(0.60–1.00)	0.048	0.79	(0.62–1.03)	0.078	0.80	(0.61–1.04)	0.090	0.79	(0.61–1.03)	0.088
Q3	0.77	(0.59–1.00)	0.049	0.77	(0.59–1.00)	0.050	0.80	(0.61–1.04)	0.095	0.78	(0.59–1.02)	0.072	0.78	(0.59–1.03)	0.082
Q4 - High weight	0.81	(0.61–1.06)	0.122	0.81	(0.61–1.06)	0.122	0.84	(0.64–1.11)	0.220	0.79	(0.60–1.05)	0.111	0.79	(0.60–1.05)	0.110
Mother smoked during pregnancy															
No	1			1			1			1			1		
Sometimes	1.60	(1.14–2.24)	0.007	1.59	(1.13–2.23)	0.008	1.51	(1.07–2.14)	0.019	1.45	(1.01–2.08)	0.043	1.41	(0.98–2.02)	0.061
Moderately	1.24	(0.97–1.59)	0.081	1.24	(0.97–1.59)	0.082	1.23	(0.96–1.58)	0.104	1.16	(0.89–1.51)	0.260	1.15	(0.88–1.49)	0.306
Heavily	1.43	(1.09–1.86)	0.009	1.42	(1.09–1.86)	0.010	1.35	(1.03–1.77)	0.030	1.22	(0.92–1.63)	0.174	1.23	(0.92–1.63)	0.162
Mother’s age at birth															
23 years or less	1			1			1			1			1		
24 to 27 years	0.64	(0.50–0.81)	<0.001	0.64	(0.50–0.82)	<0.001	0.67	(0.52–0.85)	0.001	0.69	(0.54–0.90)	0.005	0.69	(0.54–0.90)	0.005
28 to 31 years	0.72	(0.55–0.94)	0.016	0.73	(0.56–0.95)	0.021	0.77	(0.58–1.01)	0.058	0.82	(0.62–1.08)	0.163	0.83	(0.62–1.10)	0.190
32 years or more	0.73	(0.55–0.96)	0.023	0.73	(0.55–0.96)	0.027	0.79	(0.59–1.05)	0.103	0.82	(0.61–1.11)	0.195	0.83	(0.62–1.12)	0.220
Breastfed															
No	1			1			1			1			1		
Yes, for less than 1 month	1.00	(0.78–1.27)	0.983	1.01	(0.79–1.28)	0.966	1.02	(0.80–1.30)	0.895	1.01	(0.78–1.30)	0.965	1.01	(0.78–1.30)	0.969
Yes, more than 1 month	1.10	(0.89–1.36)	0.386	1.11	(0.90–1.38)	0.330	1.15	(0.92–1.42)	0.212	1.15	(0.92–1.43)	0.231	1.16	(0.93–1.45)	0.194
Parity															
Oldest child	1			1			1			1			1		
2nd child	0.88	(0.71–1.10)	0.260	0.88	(0.70–1.09)	0.239	0.86	(0.68–1.07)	0.180	0.89	(0.70–1.12)	0.309	0.88	(0.70–1.12)	0.300
3rd child or more	0.87	(0.68–1.11)	0.273	0.86	(0.67–1.10)	0.227	0.80	(0.62–1.04)	0.097	0.83	(0.63–1.08)	0.158	0.81	(0.62–1.06)	0.133
Gestational age															
38 weeks or less	1			1			1			1			1		
39 to 41 weeks	0.83	(0.59–1.15)	0.266	0.84	(0.60–1.17)	0.294	0.83	(0.59–1.16)	0.277	0.88	(0.62–1.23)	0.449	0.87	(0.62–1.23)	0.435
42 weeks or more	0.89	(0.62–1.26)	0.505	0.89	(0.63–1.27)	0.527	0.88	(0.61–1.26)	0.482	0.91	(0.63–1.32)	0.629	0.91	(0.63–1.31)	0.616
Type of delivery															
Vaginal	1			1			1			1			1		
Emergency caesarean	1.79	(0.91–3.52)	0.092	1.81	(0.92–3.56)	0.087	1.85	(0.93–3.65)	0.078	1.74	(0.85–3.56)	0.128	1.79	(0.87–3.66)	0.112
Elective caesarean	0.92	(0.37–2.31)	0.865	0.93	(0.37–2.32)	0.875	0.97	(0.39–2.44)	0.956	0.93	(0.36–2.37)	0.875	0.94	(0.37–2.40)	0.894
Adverse childhood experiences															
None				1			1			1			1		
One				1.19	(0.96–1.48)	0.106	1.15	(0.93–1.43)	0.194	1.15	(0.92–1.44)	0.222	1.12	(0.90–1.41)	0.314
Two or more				1.07	(0.76–1.52)	0.695	1.01	(0.71–1.45)	0.942	0.96	(0.66–1.39)	0.836	0.94	(0.65–1.36)	0.735
Smoking status at 23															
Non-smoker							1			1			1		
Former smoker							0.81	(0.64–1.04)	0.097	0.83	(0.64–1.07)	0.155	0.83	(0.64–1.08)	0.173
Smoker - Less than 10 cig.							0.92	(0.65–1.30)	0.628	0.95	(0.67–1.36)	0.797	0.95	(0.66–1.35)	0.763
Smoker - 10 to 19 cig.							0.88	(0.65–1.19)	0.409	0.97	(0.71–1.33)	0.860	0.94	(0.69–1.28)	0.694
Smoker - More than 20 cig.							1.08	(0.81–1.44)	0.602	1.14	(0.85–1.53)	0.393	1.09	(0.81–1.47)	0.555
Alcohol consumption at 23															
Moderate							1			1			1		
Abstainers							0.98	(0.79–1.21)	0.846	0.91	(0.73–1.13)	0.384	0.87	(0.70–1.09)	0.230
Heavy drinkers							0.80	(0.58–1.10)	0.174	0.75	(0.54–1.04)	0.089	0.75	(0.54–1.05)	0.093
Physical activity at 23															
Physically active							1			1			1		
Moderately active							0.84	(0.60–1.18)	0.309	0.83	(0.58–1.17)	0.281	0.83	(0.59–1.18)	0.306
Inactive							1.18	(0.93–1.50)	0.167	1.12	(0.88–1.44)	0.354	1.10	(0.85–1.41)	0.472
Malaise inventory at 23															
No							1			1			1		
Yes							1.25	(0.91–1.72)	0.161	1.19	(0.86–1.66)	0.300	1.17	(0.84–1.64)	0.346
Education level at 23															
Passed A levels							1			1			1		
Passed O levels							1.11	(0.84–1.46)	0.455	1.05	(0.79–1.40)	0.723	1.01	(0.76–1.35)	0.950
No qualifications							1.23	(0.90–1.69)	0.191	1.08	(0.78–1.50)	0.632	0.97	(0.69–1.37)	0.869
BMI at 23															
Normal										1			1		
Underweight										0.41	(0.22–0.74)	0.003	0.41	(0.23–0.75)	0.004
Overweight										2.71	(2.08–3.52)	<0.001	2.63	(2.02–3.44)	<0.001
Obese										7.82	(4.63–13.21)	<0.001	7.41	(4.39–12.51)	<0.001
Occupational social class at 33															
Non-manual													1		
Manual													1.09	(0.85–1.40)	0.487
Wealth at 33															
Not owner													1		
Owner - Q1 (Low price)													0.95	(0.71 - 1.27)	0.722
Owner - Q2													0.83	(0.61–1.13)	0.240
Owner - Q3													0.74	(0.54–1.03)	0.072
Owner - Q4													0.72	(0.50–1.03)	0.076
Marital status at 33															
Couple													1		
Single													0.92	(0.64–1.32)	0.646
Divorced or widowed													1.06	(0.75–1.52)	0.728

Model 1 included mother’s pre-pregnancy BMI and all variables measured at or around birth; Model 2: Model 1 + ACE; Model 3: Model 2 + potential mediators measured at 23 years except for participants’ BMI at 23 years; Model 4: Model 3 + participant’s BMI at 23 years; Model 5: Full model

Regarding the association between ACE and MetS, the association between being exposed to at least two adversities and MetS decreased by 45.5 % (95 % CI: 13.3–217.9 %) after inclusion of perinatal confounders compared with crude association and was no longer statistically significant (OR = 1.19, 95 % CI: 0.89–1.59).

Lower socioeconomic position during childhood, emergency caesarean delivery, being physically inactive and not a home owner during adulthood were independently associated with MetS.

In women (Table 4, Fig. 2b) the link between mother’s pre-pregnancy BMI and MetS persisted after adjustment for perinatal confounders (model 1), as well as after inclusion of ACE (model 2). The inclusion of potential mediators at 23 years did not change the strength of this association (model 3) except for when participants’ BMI was added (model 4). Compared with model 1, after adding participant’s BMI at 23 years, the association between mother’s pre-pregnancy BMI and MetS was attenuated by 44.6 % (95 % CI: 10.2–154.7 %) for obese (OR = 1.43, 95 % CI: 0.92–2.22) and by 25.3 % (95 % CI: 5.1–57.9 %) for overweight mothers before pregnancy (OR = 1.38, 95 % CI: 1.10–1.72) respectively. The inclusion of variables at 33 years (model 5) did not alter the association.

Regarding the association between ACE and MetS, the association between being exposed to one adversity and MetS decreased by 27.3 % (95 % CI: 2.2–122.1 %) after inclusion of perinatal confounders compared with crude association and was no longer statistically significant (OR = 1.19, 95 % CI: 0.96–1.48).

Lower socioeconomic position during childhood, emergency caesarean delivery, low birthweight, mothers younger than 23 years old at birth, smoking during pregnancy, not being a home owner during early adulthood were independently associated with MetS.

Model 1 included all variables measured at or around birth; Model 2: Model 1 + ACE; Model 3: Model 2 + potential mediators measured at 23 years except for participants’ BMI at 23 years; Model 4: Model 3 + participant’s BMI at 23 years; Model 5: Full model.

All models were also run on complete cases (1876 men and 1922 women). No significant changes regarding significant variables or direction of associations were found (data not shown).

## Discussion

This study sets out a comprehensive lifecourse approach to examine the influence of both the early nutritional environment, and the psychosocial environment, on the subsequent risk of MetS. By taking a lifecourse approach, many mediating factors that may explain pathways between early nutrition and early psychosocial stress and subsequent MetS are examined. The main finding from our study is that both early nutritional and psychosocial environments are independently associated with subsequent risk of MetS. Mother’s pre-pregnancy BMI was associated with MetS in mid-life after taking into account a large range of confounders and three potential pathways (socioeconomic/materialist, psychological and behavioral). The association appeared to be mediated to a sizeable extent by offspring’s BMI at age 23, suggesting mechanisms via a mother to child transmission of BMI. However, participants’ BMI at age 23 did not fully explain the link between mother’s pre-pregnancy BMI and MetS suggesting that unidentified mechanisms may be operating other than those captured through the confounding and mediating variables used in this study, specifically health behaviors and socioeconomic pathways. The role of ACE was not clear. The positive association between ACE and MetS was indeed not significant after taking early life confounders into account, in particular childhood socioeconomic conditions and birth conditions that were independently associated with risk of MetS.

The main strength of this study is that data were prospectively measured and the large number of confounders and mediators we consider, which made that this study includes a large panel of confounders and mediators to explore MetS in a lifecourse perspective. Consequently, we were able to analyze both early nutritional and psychosocial exposures that are mainly studied separately in the literature and also to consider early birth conditions that are largely not taken into account in studies on MetS. There are also a number of limitations that need to be considered, however. The first is regarding the definition of MetS we used in the study. The definition of MetS has varied over the past decade. The prevalence of MetS may be lower when using definitions other than NCEP-ATPIII. However the risks of cardiovascular events, diabetes mellitus and hypertension are similar for NCEP-ATPIII and American Heart Association or International Diabetes Federation definitions [45]. Glycaemia was not collected in the biomedical survey used here therefore we used HbA1c with a cut-off above 6.5 % to define hyperglycaemia. HbA1c has been defined as a marker to identify diabetic status and used in other studies instead of glycaemia [46]. We were also unable to take into account whether people were treated for hypertension, diabetes or high cholesterol level because accurate data were not available. Our definition of MetS is thus a conservative one. Another weakness is the amount of missing data caused by attrition in the cohort. This weakness is partly addressed by the use of multiple imputations in our models. The standard application of multiple imputation assumes that data are MAR meaning that the probability of missing data depends on the observed data but not the missing data, which is an unverifiable assumption. Thus we cannot rule out that some data are Missing Not At Random. A way to render the MAR assumption more plausible and to limit the impact of missing not at random missingness is to include a large numbers of covariates in multiple imputations. In our analyses all important causes of missingness such as demographic characteristics, socio–economic position indicators and behavioral variables are included in the models. Any other missingness that is not accounted for by these variables is assumed to be completely random since all major systematic causes of missingness have been accounted for. We believe that this is a reasonable assumption since it has been shown that socio–economic position and age and are the main drivers of attrition in population surveys in the UK [47, 48]. Furthermore, analyses on complete cases gave a similar result which increases the robustness of our findings. Another limitation concerns the NCDS recruitment that was done in March 1958. So we do not take account for potential effects of birth-month variations that may influence the neuroendocrine regulation of metabolism and obesity [49]. Other potential limitations concern the analyses. To illustrate the influence of confounders and mediators on the associations between our two main exposures and MetS, we calculated B attenuation after inclusion of potential confounders and mediators. This approach is not a formal mediation analysis and may provide biased estimates, however when controlling for potential mediator-outcome confounders, as we did here by considering a large range of variables in analyses, the risk of bias is limited [41]. We also used a bootstrap method to improve the precision of the percent attenuation. Our use of health behaviors at 23 years as a proxy for behavioral patterns in early adulthood is also a limitation. Adult health behaviors were collected in the cohort at ages 23, 33, 41, 46. Constructing health behavior pathways using repeated measures, may explain a significantly greater part of the association between mother’s pre-pregnancy BMI and MetS compared to a baseline-only assessment of behaviors, as observed elsewhere [43]. However the inclusion of behaviors in the model did not change the association between maternal BMI before pregnancy and MetS except for BMI. So it is not likely that behaviors even by considering them in a longitudinal way, explain a significant part of the association between maternal BMI before pregnancy and MetS. Finally, it is possible that we omitted important confounders or mediators in our models. It would have been optimal to control for the mother’s gestational diabetes status, to control for an intrauterine exposure to hyperglycemia, however accurate information on this was not available. By including birthweight we should be able to capture any macrosomia effects of gestational diabetes on the cohort member. Moreover, some of the measures we used may be susceptible to measurement error, like self-reported data on maternal weight that may have resulted in some misclassification, even if self-reported weight is generally accurate in validity studies [50].

We found that mother’s pre-pregnancy BMI may be an important determinant of metabolic pathology in mid-life, mainly operating via a mother-child body-size transmission pathway. Previous works have shown that children exposed to maternal obesity in early life presented higher levels of some MetS components [17]. In our study this association was mainly attenuated after including offspring’s BMI at 23 years in the model, suggesting that this association could be explained in a significant part by a mother to child transmission of BMI. An intergenerational adiposity association is a widely observed phenomenon [16, 51–53]. One of the main issues is to explain underlying pathways and notably the respective contributions of shared environments and genetic factors in this transmission. Some studies have observed a similar influence of mother and father’s BMI on the subsequent risk of obesity in offspring [52]. Such results may point towards an influence of shared family characteristics, such as diet and physical activity. However studies that included such mediators concluded that the mother to child transmission of BMI was attenuated but still persisted after taking adult health behaviors, including diet, or socioeconomic factors into account [16, 51]. In fact, the intrauterine environment may have an important role to play [9]. Offspring exposed to maternal hyperglycaemia during their intrauterine development are more at risk of metabolic disorders such as obesity in adulthood, and that changes in maternal nutrition, in particular during fetal development, may influence subsequent predisposition to the MetS [9, 19]. Such results are in accordance with the fetal overnutrition hypothesis or an early life nutritional programming of the metabolic syndrome, a phenomenon that has been shown in animals [18]. To investigate this hypothesis in humans the majority of studies use birthweight as a proxy of intrauterine exposures during pregnancy. In our study we consider birth and perinatal conditions, in particular birthweight, and the inclusion of such variables in models did not significantly change the association between mother’s pre-pregnancy BMI and MetS in offspring. Unfortunately, in our study, we had no information on mothers’ diet during pregnancy and after birth, no information on the way babies were fed except breastfeeding and no information on diet during childhood. The information collected on mother’s pre-pregnancy BMI, however, offers an opportunity for us to capture through a proxy variable something of cohort members’ early life nutritional environment of cohort members. Ideally, we would have liked to have information on weight gain during pregnancy or parents’ waist circumferences before pregnancy. However such data are not available in the NCDS cohort. The parent–child transmission of adiposity deserves further investigation using more relevant and detailed data to characterize both nutritional environment around pregnancy and early life, and mothers’ metabolic status. With this in mind, studies analyzing the effect of mother and father adiposity on offspring adiposity at different stages of life are needed.

We found no association between early ACE and MetS after adjusting for confounders such as early socioeconomic and birth conditions. Few previous studies have observed an influence of ACE by using retrospective measures for ACE [24, 54] or using both retrospective and prospective measures [30]. There is strong evidence for the role of stress in the long term etiology of cardiometabolic diseases [31]. This evidence leads to questions about the measures used to capture psychosocial stress and specifically our definition of adversity. Thomas et al. [30] have shown that the link between adversity and subsequent risk of obesity and type 2 diabetes in mid-life was different according to the definition used for adversity suggesting that we can be exposed to measurement error or that different measurements of ACE may be capturing different types of stress. In particular we did not use any variables on sexual or physical abuse that has been shown as associated with obesity [30], because these variables were retrospectively measured (at the same time as MetS was measured) as opposed to our definition which used only variables measured prospectively to avoid recall bias. Of course, childhood adversity may not be the only or best measure of stress in early life, both prenatal and postnatal periods could also a phase where early life stress may be picked-up. For example, maternal stress during pregnancy has been associated with central adiposity in children at 13 years [55]. In animals, prenatal stress has been associated with metabolic disturbances in adult offspring [56]. As we previously observed [38], emergency caesareans were associated with subsequent risk of MetS in the NCDS cohort. Recent reviews have shown that caesarean section was a risk factor of adult obesity [57, 58]. The main hypothesis refers to the nature of the offspring microbiome that could differ according the mode of delivery [59]. In our study, it was not caesarean sections per se that influenced the risk of MetS but only if they were carried-out in an emergency. Emergency caesareans may act as a proxy of perinatal stressful events that may play a role in subsequent risk of MetS. Other perinatal variables may also be proxies for early life stress [54, 60], like birthweight or smoking during pregnancy that were associated with risk of MetS in our study, in particular among women. It is noteworthy that the influence of these ‘perinatal stress’ proxies appear stronger in women than in men suggesting than women could be more sensitive to early life stress exposures than men in accordance with other studies. Early life exposures have indeed been found to be more strongly associated with autonomic nervous system reactivity in women [60]. Regarding risk of MetS, different biological and social pathways have been identified in men and women [13, 44].

Psychosocial stress is also associated with socioeconomic conditions [25] which are known to be associated with subsequent health and in particular with MetS [12, 14, 61]. In our study, psychosocial adversity was not significant after including socioeconomic variables such as mother’s level of education and father’s social class at birth which were independent risk factors of MetS. The fact that the link between childhood socioeconomic conditions and MetS persisted in the full model, after the inclusion of a large range of confounders and mediators, questions the mechanisms involved and not considered in the study. Pathways likely to explain this association deserve to be explored in more depth.

## Conclusion

Our results show that it is relevant to study MetS taking a lifecourse perspective. Indeed mother’s BMI before pregnancy is strongly associated the likelihood of MetS in adulthood suggesting that some aspects of early nutritional environment may influence the risk of subsequent MetS in midlife. One of the main mechanisms may involve a mother-to-child BMI transmission which deserves more attention. Other mechanisms linking mother’s pre-pregnancy BMI and MetS are likely to be operational. The link between exposure to adversity during childhood and the risk of MetS is less clear since this association was not significant after considering early socioeconomic and birth conditions that were independently associated with risk of MetS. Pathways linking early psychosocial stress, by disentangling the various aspects and dimensions of this stress and subsequent risk of MetS deserve further investigations. These results can have potentially important implications for prevention policies targeting MetS, by identifying early life and the parent to child transmission of adiposity as possible targets. The implementation of potential prevention policies will need to understand how such transmissions occur.

## Abbreviations

ACE: Adverse childhood experiences
BMI: Body mass index
HbA1c: Glycosylated hemoglobin
MAR: Missing at random
MetS: Metabolic syndrome
NCDS: National Child Development Study
NCEP-ATP III: National Cholesterol Education Program Adult Treatment Panel III
